# Effect of Finishing Protocol Treatments on Optical Properties of Super Translucent Zirconia After Simulated Wear

**DOI:** 10.3390/dj14010003

**Published:** 2025-12-20

**Authors:** Maja Žagar, Ines Kovačić, Robert Pongrac, Robert Ćelić

**Affiliations:** 1 Department of Removable Prosthodontics, School of Dental Medicine, University of Zagreb, 10000 Zagreb, Croatia; mpavic@sfzg.hr (M.Ž.); kovacic@sfzg.hr (I.K.); 2 Dental Laboratory Robert Pongrac, Odra, 10000 Zagreb, Croatia; info@robertpongrac.com; 3 Clinical Hospital Center Zagreb, 10000 Zagreb, Croatia

**Keywords:** super translucent zirconia, color difference, translucency parameter, spectrophotometer, wear

## Abstract

**Background/Objectives**: Super translucent zirconia (ST zirconia) is increasingly used for esthetic restorations, but its optical stability after mechanical wear remains unclear. This study aimed to evaluate the effects of three finishing protocols—polishing, glazing, and staining followed by glazing (Chroma + Glaze)—on the color stability and translucency of ST zirconia after simulated toothbrushing. **Methods**: Sixty zirconia specimens (Vita YZ ST, shade A1) were fabricated and divided into three groups, namely Polished, Glazed, and Chroma + Glaze (*n* = 20 in each group). Color (L*, a*, b*) was measured using a spectrophotometer (VITA Easyshade V; VITA Zahnfabrik) before and after 10,000 brushing cycles (200 g load, 1.5 Hz). Translucency parameters (TP_ab_, TP_00_) were calculated, while color changes were assessed using CIELAB (ΔE_ab_) and CIEDE2000 (ΔE_00_) formulas. Data were analyzed using two-way ANOVA and Kruskal–Wallis tests (*α* = 0.05). **Results**: Treatment significantly affected translucency (H = 46.79, *p* < 0.001; H = 21.09, *p* < 0.001), indicating consistent differences among the three treatment groups. Bonferroni-adjusted post hoc comparisons showed that Chroma + Glaze exhibited significantly lower TP_00_ values than Glaze in both measurements (*p* < 0.0001; *p* < 0.001), as well as lower values than Polished in both measurements (*p* < 0.0001; *p* = 0.0147, respectively). Kruskal–Wallis analysis revealed significant differences among finishing protocol groups for both ΔE_ab_ (H = 13.21, *p* < 0.0014) and ΔE_00_ (H = 9.14, *p* = 0.0104), with Chroma + Glaze exhibiting the smallest ΔE values (ΔE_00_ ≈ 0.33) below the perceptibility threshold. **Conclusions**: The finishing protocol significantly influences the optical behavior of ST zirconia after simulated wear. The Chroma + Glaze group demonstrated the highest color stability and lowest translucency, suggesting enhanced long-term esthetic performance. In contrast, polished zirconia showed greater color variation and an increase in translucency, indicating lower optical stability under brushing abrasion.

## 1. Introduction

Monolithic multilayer zirconia has revolutionized restorative dentistry by offering high mechanical strength, biocompatibility, and suitability for computer-aided design and computer-aided manufacturing (CAD-CAM) fabrication [1,2,3]. In recent years, advancements in material science have led to the development of high-translucency zirconia intended for esthetically demanding clinical cases, particularly in the anterior region [4]. Super translucent zirconia (ST zirconia) attempts to bridge the gap between strength and esthetics, which was traditionally fulfilled by glass ceramics like lithium disilicate.

The color and translucency of a dental restoration are critical for achieving patient satisfaction, particularly in anterior teeth, where there are more esthetic demands [5]. The finishing protocol, such as polishing, glazing, and staining followed by glazing, are commonly used to enhance the visual appeal of monolithic zirconia restorations [6,7]. These treatments affect surface roughness, gloss, and ultimately the material’s optical properties, including color parameters (L*, a*, b*, C*, h) and translucency. Previous studies have confirmed that surface gloss and texture can influence how the color and translucency of dental ceramics are perceived, making the finishing protocol an essential consideration in restorations [8,9]. However, while these techniques improve immediate esthetics, their long-term effect on color stability and translucency under mechanical wear, such as toothbrushing abrasion, is not fully understood.

Color stability, commonly defined as color difference (ΔE) in the CIEDE2000 system, is critical for dental restorations. Zirconia is often regarded as color-stable, but optical changes can occur with the finishing protocol and wear [10]. For example, a study simulating 15 years of brushing reported color difference (ΔE) values around 1.5—below the clinical perceptibility threshold (~2.6). Nevertheless, glazed specimens exhibited less color change than polished ones, indicating a protective effect of glazing against chromatic shifts [11]. In addition, the translucency parameter (TP)—calculated as the difference in color between specimens on white vs. black backgrounds—provides a quantitative measure of light transmission. Recent meta-analyses show that higher yttria content correlates with an increased TP, although glazing and thickness effects may obscure these differences. Thick glaze layers slightly reduce the TP in some systems, but effects vary among brands [10].

Previous research has shown that brushing abrasion can alter the surface gloss and roughness of zirconia, potentially affecting its optical properties [12]. Simulated toothbrushing protocols are frequently used to evaluate the mechanical stability of dental materials under conditions resembling clinical wear, acknowledging that in vivo factors such as saliva, pH fluctuations, and dietary habits can also influence the wear and optical properties of zirconia restorations. Despite the widespread use of zirconia in restorative dentistry, there is a lack of studies evaluating its long-term optical stability under mechanical wear. Understanding how surface treatments affect color and translucency over time remains an important yet underexplored research gap.

This study aims to fill this gap by systematically evaluating the influence of three common finishing protocols—polishing, glazing, and staining followed by glazing—on optical properties such color differences (ΔE) and the translucency parameter (TP) of super translucent monolithic zirconia before and after simulated toothbrushing wear. Such information is crucial for clinicians seeking to balance esthetic outcomes with long-term stability when selecting the finishing protocol for monolithic zirconia restorations.

## 2. Materials and Methods

### 2.1. Specimen Preparation

A total of sixty rectangular specimens were designed and fabricated from a super translucent monolithic zirconia disk in A1 shade (Vita YZ ST, shade A1; VITA Zahnfabrik, Bad Säckingen, Germany). Each specimen was designed with dimensions of 7 mm × 11 mm × 2 mm. The samples were milled using a CAD/CAM system and sintered at 1530 °C (VITA SMART.FIRE ADVANCED, Vita Zahnfabrik, Bad Säckingen, Germany) in accordance with the manufacturer’s recommendations. The thickness of all specimens was verified using a digital caliper (±0.01 mm accuracy) to ensure standardization. Specimens outside the mentioned thicknesses were replaced.

Following sintering, the specimens were randomly divided into three groups (*n* = 20) according to the finishing protocol applied—Polished (P), Glazed (G), and Chroma + Glaze (CG). The Polished (P) specimens were polished using a zirconia polisher (ZrO_2_ Polishers; Komet Dental) and a polishing paste (Polish All-in-One Paste; Renfert, Hilzingen, Germany) for two minutes. For the Glazed (G) group, a layer of glaze (Vita Lumex Unique 370 glaze; VITA Zahnfabrik, Bad Säckingen, Germany) was applied to the surface and fired under a vacuum at 750 °C for one minute, followed by drying for six minutes with a temperature decrease of 50 °C per minute. In the Chroma + Glaze (CG) group, a chroma stain (Vita Lumex Unique 330 Red-Brown Chroma A; VITA Zahnfabrik, Bad Säckingen, Germany) was applied, followed by a glaze layer (Vita Lumex AC 370 glaze; VITA Zahnfabrik, Bad Säckingen, Germany), with no additional polishing afterwards. The staining and glazing procedures were performed using the same firing protocol as described for the glazed specimens. All the specimens were prepared by a single highly trained dental technician.

### 2.2. Sample Size Justification

The sample size was determined a priori using G*Power software (version 3.1, Universität Düsseldorf, Germany) based on data from previous optical studies on zirconia. For a mixed-model ANOVA with three treatment groups and two repeated measures (Before/After), assuming a medium effect size (f = 0.25), α = 0.05, and desired power (1 − β) = 0.80, a minimum total of 18 specimens was required. Accordingly, 60 zirconia plates (*n* = 20 per group) were fabricated to ensure adequate statistical power.

### 2.3. Color and Translucency Measurements

Color measurements were performed using a calibrated spectrophotometer (VITA Easyshade V; VITA Zahnfabrik, Bad Säckingen, Germany) under standardized lighting conditions by one trained operator. The spectrophotometer was recalibrated before each set of measurements. Each specimen was measured against both white and black backgrounds to evaluate color and translucency. The CIE Lab* values were recorded three times for each specimen, and mean values were used for analysis. From these values, color differences (ΔE) and the translucency parameter (TP) of the same specimen before vs. after brushing wear were calculated. The color difference (ΔE) was calculated using the CIELAB (ΔEab) [13,14] and CIEDE2000 (ΔE_00_) [15] formula between baseline and post-wear measurements, while the translucency parameter (TP) was calculated as the color difference between measurements taken on white and black backgrounds, before and after brushing wear.

CIELAB formula:

$$\Delta E_{\left\{ab\right\}}=\;\sqrt{\left\{{\left(\Delta L^{*}\right)}^{2}+\;{\left(\Delta a^{*}\right)}^{2}+\;{\left(\Delta b^{*}\right)}^{2}\right\}}$$

CIEDE2000 formula:

$$\Delta E_{00}={\left[{\left(\frac{\Delta L^{\prime }}{K_{L}S_{L}}\right)}^{2}+{\left(\frac{\Delta C^{\prime }}{K_{C}S_{C}}\right)}^{2}+{\left(\frac{\Delta H^{\prime }}{K_{H}S_{H}}\right)}^{2}+R_{T}\left(\frac{\Delta C^{\prime }}{K_{C}S_{C}}\right)\left(\frac{\Delta H^{\prime }}{K_{H}S_{H}}\right)\right]}^{1/2}$$

Translucency parameter formula—TP_ab_:

$$TP_{ab}={\left[{\left(L_{W}-L_{B}\right)}^{2}+{\left(a_{W}-a_{B}\right)}^{2}+{\left(b_{W}-b_{B}\right)}^{2}\right]}^{\left(1/2\right)}$$

Translucency parameter formula—TP_00_:

$${TP}_{00}={\left[{\left(\frac{L_{B}^{\prime }-L_{W}^{\prime }}{k_{L}S_{L}}\right)}^{2}+{\left(\frac{C_{B}^{\prime }-C_{W}^{\prime }}{k_{C}S_{C}}\right)}^{2}+{\left(\frac{H_{B}^{\prime }-H_{W}^{\prime }}{k_{H}S_{H}}\right)}^{2}+R_{T}\left(\frac{C_{B}^{\prime }-C_{W}^{\prime }}{k_{C}S_{C}}\right)\left(\frac{H_{B}^{\prime }-H_{W}^{\prime }}{k_{H}S_{H}}\right)\right]}^{\frac{1}{2}}$$

### 2.4. Simulated Toothbrushing Wear

**Figure 1:**
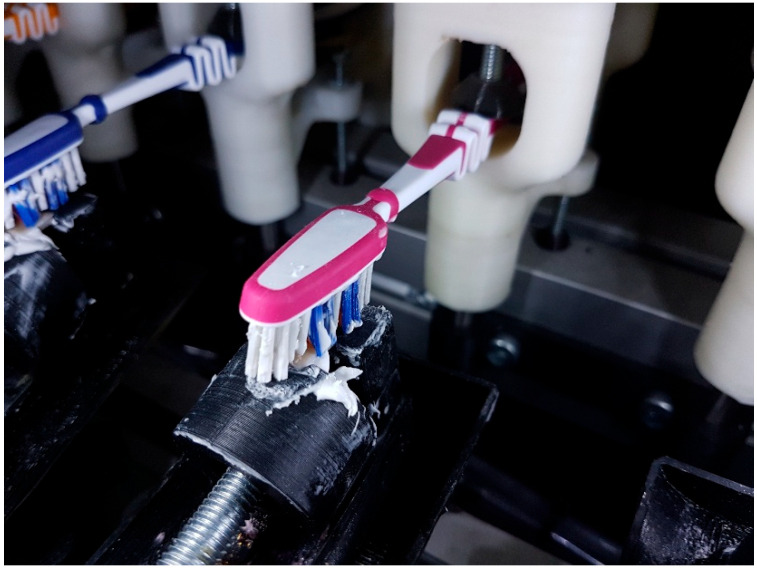
Brushing was performed using a custom-built mechanical brushing simulator.

All specimens were subjected to a simulated toothbrushing protocol to replicate one year of clinical oral hygiene. Brushing was performed using a custom-built mechanical brushing simulator (Figure 1). Each specimen underwent 10,000 brushing cycles with medium-bristle toothbrush heads (Aquafresh Clean & Flex medium, GLAXOSMITHKLINE, Budapest, Hungary) and a standard toothpaste (Aquafresh Active Fresh toothpaste, GLAXOSMITHKLINE, Hungary). A constant load of 200 g was applied during brushing at a frequency of 1.5 Hz. After brushing, specimens were rinsed with deionized water, air-dried, and re-evaluated using the same color and translucency measurement protocol described above.

### 2.5. Statistical Analysis

Data distribution normality was assessed using the Shapiro–Wilk test for all quantitative variables, including TP_ab_, TP_00_, ΔE_ab_, and ΔE_00_. All variables demonstrated significant deviations from normality (*p* < 0.001). Consequently, non-parametric statistical methods were applied throughout.

For translucency parameters (TP_ab_ and TP_00_), comparisons between the three finishing protocols (Polished, Glazed, Chroma + Glaze) were performed using the Kruskal–Wallis test, followed by pairwise Mann–Whitney U tests with Bonferroni correction when overall significance was detected. Differences between phases (before vs. after brushing) within each treatment group were analyzed using the Wilcoxon signed-rank test.

For the color difference outcomes (ΔE_ab_ and ΔE_00_), data were non-normally distributed as well; therefore, Kruskal–Wallis tests were used to compare the three finishing protocols (Polished, Glazed, Chroma + Glaze). When significant, pairwise differences were assessed using Dunn’s post hoc test with Bonferroni adjustment. Statistical significance was set to α = 0.05.

## 3. Results

### Translucency Parameter

**Figure 2:**
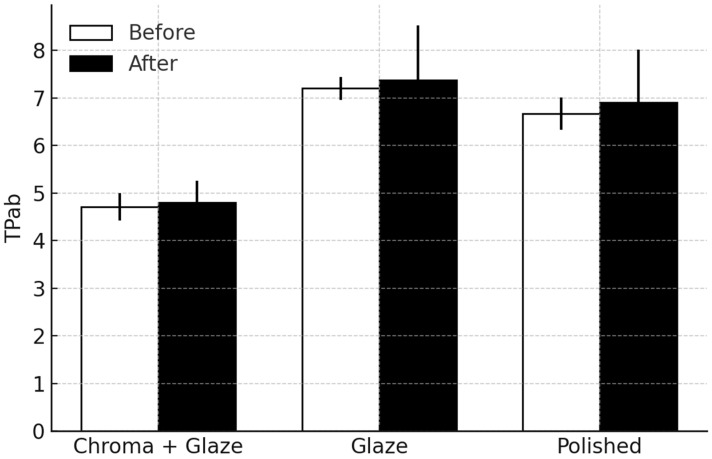
Median values with quartiles for the translucency parameter (TP_ab_) by treatment and phase (before or after simulated wear).

Descriptive statistics for the translucency parameter (TP_ab_) are presented in Figure 2 and Table 1. Kruskal–Wallis analysis demonstrated a significant effect of the finishing protocol in the measurements performed before and after brushing (H = 43.48, *p* < 0.001; H = 18.67, *p* < 0.001, respectively), indicating meaningful differences among the Polished, Glazed, and Chroma + Glaze specimens across both phases.

Pairwise Mann–Whitney U comparisons with Bonferroni adjustment (Table 1) confirmed that Chroma + Glaze exhibited significantly lower TP_ab_ values than Glazed in both measurements (*p* < 0.0001 and *p* < 0.001, respectively), as well as significantly lower values compared with Polished (*p* < 0.0001 and *p* = 0.0152, respectively). No significant differences were found between Glazed and Polished in either phase (*p* > 0.20).

Wilcoxon signed-rank tests showed no significant within-treatment differences between both measurements (all *p* > 0.64), indicating that brushing did not produce measurable changes in TP_ab_.

**Figure 3:**
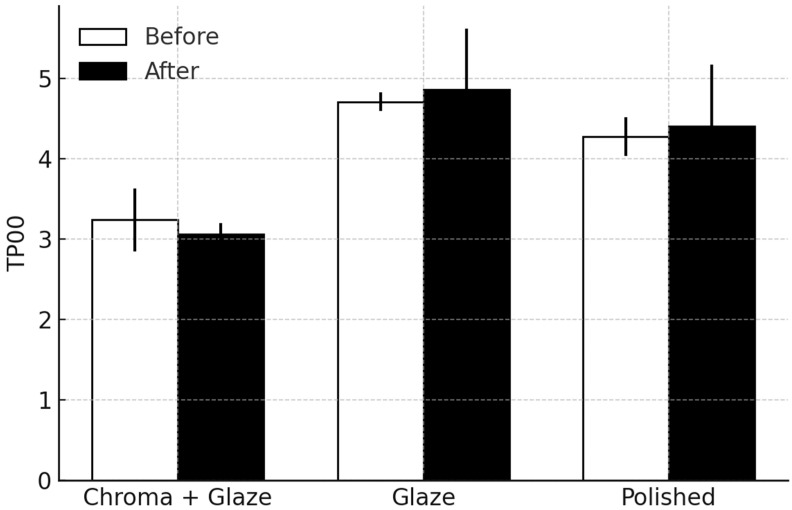
Median values with quartiles for the translucency parameter (TP_00_) by treatment and phase (before or after simulated wear).

Descriptive statistics for TP_00_ are summarized in Figure 3 and Table 2.

Kruskal–Wallis tests demonstrated a significant effect of the finishing protocol in the measurements performed before and after brushing (H = 46.79, *p* < 0.001; H = 21.09, *p* < 0.001, respectively), indicating consistent differences among the three treatment groups.

Bonferroni-adjusted post hoc comparisons showed that Chroma + Glaze exhibited significantly lower TP_00_ values than Glazed in both measurements (*p* < 0.0001; *p* < 0.001, respectively), as well as also lower values than Polished in both measurements (*p* < 0.0001; *p* = 0.0147, respectively). No significant differences were observed between Glazed and Polished in either measurement (*p* > 0.20).

Wilcoxon signed-rank tests showed that none of the treatment groups displayed significant changes between the two measurements (all *p* > 0.64), indicating that the brushing procedure did not meaningfully affect TP_00_.

## 4. Color Difference

**Figure 4:**
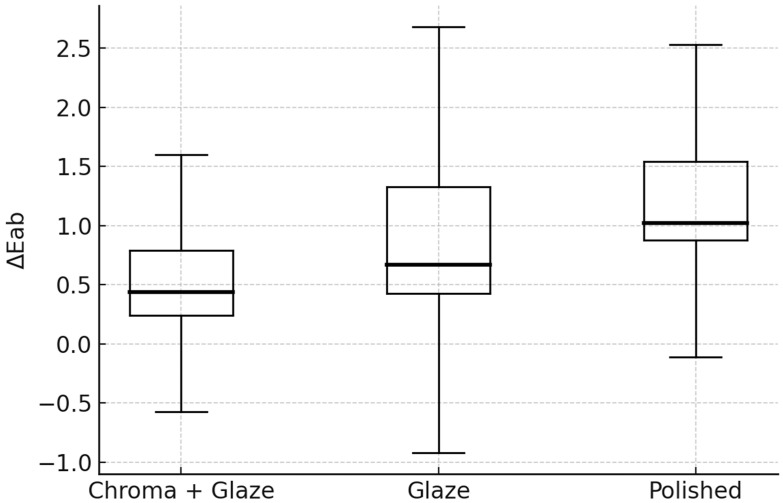
Median values with quartiles for color differences (ΔE_ab_) by treatment (CIELAB formula).

**Figure 5:**
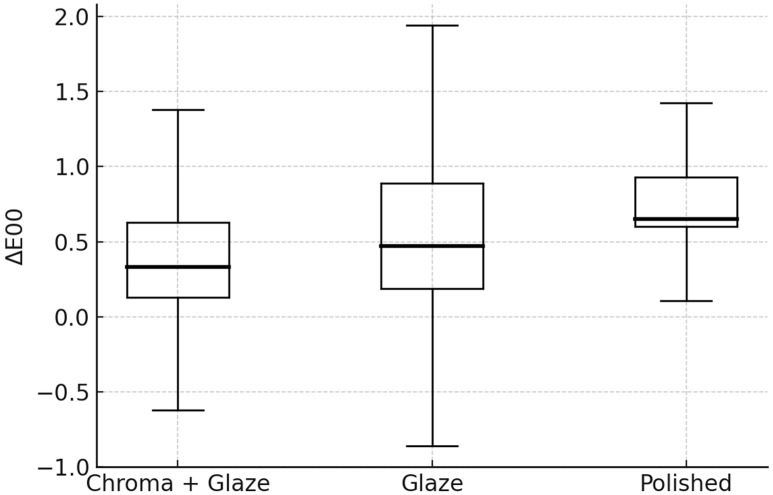
Median values with quartiles for color differences (ΔE_00_) by treatment (CIELAB formula).

Descriptive statistics for color differences according to the CIELAB (ΔE_ab_) formula are presented in Figure 4 and Table 3, while statistics according to the CIEDE2000 (ΔE_00_) formula are presented in Figure 5 and Table 4.

The Kruskal–Wallis test revealed statistically significant differences among the finishing protocol groups for both ΔE_ab_ (H = 13.21, *p* < 0.0014) and ΔE_00_ (H = 9.14, *p* = 0.0104).

Dunn-type post hoc comparisons with Bonferroni correction demonstrated that the Chroma + Glaze group exhibited significantly lower ΔE_ab_ and ΔE_00_ values compared with the Polished group (adjusted *p* = 0.00059 and *p* = 0.0076, respectively) (Table 5 and Table 6). No significant differences were observed between Chroma + Glaze and Glazed (adjusted *p* = 0.359 for ΔE_ab_; *p* = 0.717 for ΔE_00_) or between Glazed and Polished (adjusted *p* = 0.215 for ΔE_ab_; *p* = 0.257 for ΔE_00_) (Table 5 and Table 6).

The Chroma + Glaze specimens demonstrated the smallest color changes, with median ΔE_00_ values around 0.33, which is below the widely accepted perceptibility threshold and therefore considered clinically imperceptible.

## 5. Discussion

The present study investigated the influence of different finishing protocols—polishing, glazing, and staining followed by glazing—on the optical properties of super translucent zirconia, specifically focusing on translucency and color stability after simulated brushing wear. The findings demonstrated that the finishing protocol significantly affected both color differences (ΔE) and the translucency parameter (TP), with stained and glazed specimens (Chroma + Glaze) showing superior color stability and lower translucency compared to the other groups. These results emphasize the importance of selecting appropriate finishing protocol techniques not only for esthetic optimization but also for ensuring long-term stability in clinical use.

Color stability, quantified via ΔE values (ΔE_ab_ and ΔE_00_), is a critical parameter for long-term esthetic success in restorative dentistry. The ΔE thresholds for perceptibility and acceptability in clinical dentistry are generally accepted to be around 1.8–2.6 and 2.25–3.7, respectively, depending on the evaluation method and lighting conditions used [16]. In this study, all mean ΔE values for all groups remained below the perceptibility threshold, with lowest values for the Chroma + Glaze group (ΔE_00_ ≈ 0.33), indicating minimal color change even after simulated wear. This aligns with findings of Manziuc et al., who reported that glazed zirconia demonstrated lower ΔE values than polished specimens after wear, suggesting that the glaze layer acts as a protective barrier against mechanical and chemical degradation [10]. The polished group demonstrated the highest ΔE values (ΔE_ab_ = 1.02), indicating color changes potentially close to the clinical acceptability threshold in some specimens. These findings agree with those of Lee et al. [11], who showed that unglazed, polished zirconia surfaces are more prone to discoloration and surface alteration following abrasion. Additionally, a systematic review confirmed that various finishing protocols, including polishing and glazing, significantly reduce the translucency and color stability of monolithic zirconia under aging, toothbrushing, and other wear conditions [17].

Interestingly, the Chroma + Glaze group consistently exhibited lower ΔE values compared to both Polished and Glazed specimens, but a statistically significant difference only existed between the Chroma + Glaze and Polished groups. This aligns with results supported by Hashemikamangar et al. [18], who suggested that applying a stain (Chroma) followed by a glaze layer may stabilize pigments within the surface layer and reduce surface porosity, thereby improving resistance to optical changes. The additional chroma layer in these specimens may also contribute to increased color masking, effectively camouflaging minor surface alterations resulting from wear. The superior color stability observed in the Chroma + Glaze group is likely due to the combined effects of both the glaze layer and the chroma stain. While the study design does not allow for the determination of the individual contribution of each component, the glaze layer likely acts as a protective barrier against mechanical wear, whereas the chroma stain may help mask minor color changes, together resulting in lower ΔE values.

The translucency parameter (TP) decreased across all finishing protocol surface treatment groups following simulated toothbrushing, indicating that mechanical abrasion can affect the light transmission properties of even highly translucent zirconia. This observation aligns with the recent findings of Lee et al. [19], who investigated the effect of simulated toothbrushing on extrinsically stained high-translucency zirconia. Although their study did not report significant changes in TP values, they did observe increased surface roughness, suggesting that surface texture changes can influence light interaction and potentially lead to alterations in translucency over time. Further supporting this, another study evaluating the color and translucency of zirconia demonstrated significant reductions in TP values after long-term mechanical and thermal aging [20]. This reinforces the idea that repeated mechanical wear combined with temperature fluctuations may compromise the optical stability of zirconia surfaces, regardless of the initial finishing protocol. Additionally, a recent study by Pool et al. [21] found that toothbrushing with both regular and whitening toothpastes induced measurable surface degradation of monolithic zirconia, including increased roughness and reduced gloss, even after polishing or glazing procedures. While the TP was not directly measured in their study, these surface changes are known to affect how light is transmitted or scattered by the material, implying that prolonged brushing can gradually diminish translucency, particularly when surface protection layers are worn away.

Altogether, the findings from the current study are in line with these newer investigations, indicating that while finishing protocols such as glazing and staining may initially enhance optical performance, their protective effects are not permanent. Mechanical abrasion from toothbrushing can progressively alter the surface microstructure and reduce translucency, potentially compromising long-term esthetics in zirconia restorations.

Among the groups, Chroma + Glaze specimens demonstrated the lowest TP values both before and after brushing (TP_ab_ = 4.71–4.8), indicating a more opaque appearance relative to the other finishing protocols. This can be attributed to the addition of chroma pigments, which absorb and scatter more light, as previously discussed by Ban [4] in the context of yttria-stabilized zirconia systems. Glazed and Polished specimens exhibited higher TP values, with polished zirconia showing the greatest increase in translucency after brushing. However, this may also be influenced by uncontrolled surface roughening due to wear. While a certain degree of translucency is desirable for mimicking natural enamel, excessive light transmission may also lead to undesirable show-through of the underlying tooth or abutment, particularly in discolored teeth or metal-core restorations. Therefore, the slightly reduced translucency observed in Chroma + Glaze specimens could be clinically beneficial in certain scenarios, particularly for masking underlying structures in the anterior zone [5].

The present findings emphasize the importance of selecting an appropriate finishing protocol for monolithic zirconia restorations based on their intended clinical location and esthetic demands. While polishing is often preferred for its simplicity and to avoid firing cycles, it may compromise long-term color stability and is more susceptible to wear-induced surface changes. Glazing improves immediate esthetics but may not provide the same resistance to wear and discoloration as a combination of staining and glazing.

In high-esthetic-demand cases, particularly in anterior restorations, the use of chroma staining followed by glazing may offer optimal results in terms of color stability and controlled translucency. Nonetheless, it is essential to consider the balance between enhanced color stability and potentially reduced translucency, especially when striving for the most lifelike optical match to natural dentition.

This study was limited by its in vitro design and the use of a single brand of super translucent zirconia and no thermocycling procedure. While simulated brushing effectively mimicked one year of clinical wear, in vivo conditions involve more complex chemical and thermal challenges, including pH fluctuations, salivary enzymes, and dietary chromogens, which may accelerate surface degradation [22,23]. Another limitation of the present study is that surface conditioning of the intaglio or bonding surface and surface roughness were not assessed. It is well known that etching zirconia with a nitric acid–hydrofluoric acid mixture alters surface morphology, and surface roughness and gloss can influence the perception of color and translucency. However, the long-term optical behavior after such chemical treatments after aging and wear has not been fully clarified [24].

Further clinical studies are necessary to confirm whether the observed trends in ΔE and TP values persist under real-life oral conditions over longer periods. Additionally, surface roughness (Ra) and gloss measurements were not included in this study. These parameters could provide deeper insights into the mechanisms behind the observed optical changes and the role of microtopographical alterations in influencing light scattering. Future studies should also explore the long-term durability of staining agents and glaze layers under thermal cycling and variable pH conditions.

## 6. Conclusions

Within the limitations of this in vitro study, it can be concluded that the finishing protocol treatment significantly affects the optical stability of super translucent zirconia after simulated wear. The Chroma + Glaze group exhibited the least color change and lowest translucency values, indicating superior color stability but increased opacity, making it particularly suitable for anterior restorations with high esthetic demands. In contrast, polished zirconia exhibited greater color changes and higher variability after brushing, suggesting lower long-term predictability for esthetic outcomes. These findings underscore the importance of selecting surface treatments based on the clinical situation and desired esthetic results, especially when using high-translucency zirconia in the esthetic zone.

## Figures and Tables

**Table 1:** Descriptive statistics for the translucency parameter (TP_ab_) by treatment and phase (before or after simulated wear).

Treatment	Phase	N	Median	Q1	Q3	IQR
**Chroma + Glaze**	Before	20	4.71	4.44	5.01	0.57
**Chroma + Glaze**	After	20	4.8	4.36	5.27	0.91
**Glazed**	Before	20	7.2	7.16	7.64	0.48
**Glazed**	After	20	7.37	5.99	8.31	2.32
**Polished**	Before	20	6.67	6.44	7.11	0.67
**Polished**	After	20	6.9	5.18	7.4	2.22

**Table 2:** Descriptive statistics for the translucency parameter (TP_00_) by treatment and phase (before or after simulated wear).

Treatment	Phase	N	Median	Q1	Q2	IQR
**Chroma + Glaze**	Before	20	3.24	2.76	3.55	0.79
**Chroma + Glaze**	After	20	3.06	2.82	3.1	0.28
**Glazed**	Before	20	4.71	4.65	4.88	0.23
**Glazed**	After	20	4.86	3.88	5.41	1.54
**Polished**	Before	20	4.28	4.15	4.63	0.48
**Polished**	After	20	4.41	3.37	4.89	1.52

**Table 3:** Descriptive statistics for color differences (ΔE_ab_) by treatment (CIELAB formula).

Treatment	N	Median	Q1	Q3	IQR
**Chroma + Glaze**	20	0.44	0.24	0.79	0.54
**Glazed**	20	0.67	0.43	1.33	0.90
**Polished**	20	1.02	0.88	1.54	0.66

**Table 4:** Descriptive statistics for color differences (ΔE_00_) by treatment (CIEDE2000 formula).

Treatment	N	Median	Q1	Q3	IQR
**Chroma + Glaze**	20	0.33	0.13	0.63	0.50
**Glazed**	20	0.47	0.19	0.89	0.70
**Polished**	20	0.65	0.60	0.93	0.33

**Table 5:** Dunn’s post hoc test with Bonferroni correction for color differences (ΔE_ab_) by treatment (CIELAB formula).

Comparison	U	*p*
**Chroma + Glaze vs. Glazed**	129.0	ns
**Chroma + Glaze vs. Polished**	12.0	0.00059
**Glazed vs. Polished**	41.0	ns

**Table 6:** Dunn’s post hoc test with Bonferroni correction for color differences (ΔE_00_) by treatment (CIEDE2000 formula).

Comparison	U	*p*
**Chroma + Glaze vs. Glazed**	150.0	ns
**Chroma + Glaze vs. Polished**	52.0	0.0076
**Glazed vs. Polished**	112.0	ns

## Data Availability

The original contributions presented in this study are included in the article. Further inquiries can be directed to the corresponding author.
